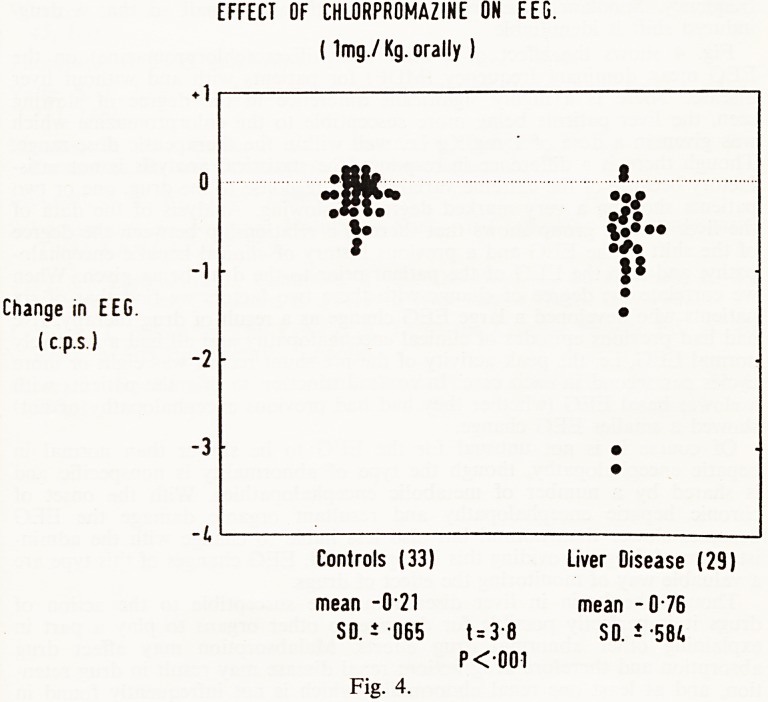# Drugs and Liver Disease

**Published:** 1969-10

**Authors:** A. E. Read


### DRUGS AND LIVER DISEASE

**A. E. Read**

I propose to review the important relationships between the liver and drug therapy, and I ask you to excuse me if my delivery is a simple one as I realize that an audience such as this is not necessarily interested in the finer points of this subject. Briefly the ways in which drugs and liver disease are involved are as follows:— Some drugs damage the liver; most drugs undergo metabolic change within the liver and their metabolism may be abnormal in liver disease; and drugs are known to have unusual and sometimes dangerous side effects in the presence of liver disease.

First I shall deal with liver disease due to drugs, which may be summarized thus:—

**Liver damage due to drugs, etc.**

| | | |
|---|---|---|
| (a) Biliary damage | canaliculus | ' CHOLESTASIS ' |
| | cholangiole | ' CHOLANGIOLITIS ' |
| (b) Liver cell | partial | JAUNDICE |
| | total | LIVER CELL FAILURE |
| (c) Other | hepatic venous obstruction, portal fibrosis/cirrhosis, neoplasia | |

The part of the liver injured and the mechanisms involved are variable, but it is convenient to classify these reactions firstly as those affecting the biliary apparatus, producing obstructive jaundice either "cholestatic" or "cholangiolitic"; secondly, as drug lesions which affect the liver cell, and which produce either liver cell jaundice or liver cell failure, depending on the intracellular site of the damage, or the extent to which whole liver cells are destroyed. An example of a drug with the former type of action is novobiacin which inhibits bilirubin conjugation at a specific intracellular site; whilst hepatic poisons and many commonly used drugs have the potentiality to injure the whole liver cell. Other parts of the microanatomy of the liver may be damaged, including the central hepatic veins and the portal zones by drugs used in the treatment of cancer but these I can refer to only in passing.

In regard to the microanatomy of the liver cell it is possible to delineate certain 'danger zones' for drugs. These include the biliary canaliculus, i.e. the commencement of the biliary apparatus; the membrane of the liver cell itself, where there can be competition for entry of bilirubin and other substances into the liver cell; and the smooth endoplasmic reticulum which is an important site of drug damage when there is interference with its function of bilirubin conjugation. These three sites may be considered singly but obviously total liver cell damage produces the disadvantages of all three lesions.

Bilirubin must be taken up into the liver cell, which involves its cleavage from its loose binding with albumin. It crosses the cell membrane—where it is in competition with other entrants—it is conjugated with glucoronide at the smooth endoplasmic reticulum (another site of drug attack) and thence becomes more polar. Suitably conjugated, it is transferred across the liver cell where again it competes with certain drugs like biliary radiological contrast materials before finally being actively excreted into the biliary canaliculus. You will see that it undergoes a hazardous journey and so perhaps it is not surprising that drug jaundice is not unusual.

At this point I should say something about the types of obstructive jaundice produced by drugs that I have called cholestatic and cholangiolitic. Their features are summarized below:

**Obstructive jaundice due to drugs**

| | Cholestatic | Cholangiolitic |
|---|---|---|
| Drug type:— | 17 alkyl-subs. steroids | phenothiazines |
| Bile retention ... ... | + | + |
| Portal infiltrate ... | − | + |
| Liver cell destruction ... | − | + |
| Systemic upset ... ... | − | + |
| Drugs idiosyncrasy ... | − | + |
| Duration ... ... | related to dose | not related to dose; can be progressive |

Not only do the drug types differ, with cholestasis being a feature particularly of the 17 alkyl-substituted testosterones and nor-testosterones, but there are differences in patient susceptibility, clinical outcome, and histology. The reason why drugs produce these disorders is unknown, but possibilities include damage to the biliary microvilli (which is unlikely), or perhaps competition between drugs and bile salts for excretion at this crucial area. Interest has centred around the possibility that certain dyhydroxy bile salts, notably lithocolic acid, may have an irritant effect on the bilary canaliculus and it is possible that cholestasis may represent damage caused by irritant bile salts. An " inflammatory " reaction related to hypersensitivity seems more likely as an explanation of the cholangiolitic lesion.

Fig. 1 summarizes the types of liver damage we have been referring to, and takes us on to consider the disorders of the liver cell, and the agents producing them. The liver cell lesions include zonal lobular damage commonly due to poisons, and a hepatitis-like lesion, both of which may on occasions produce the highly fatal type of liver disease, acute massive necrosis.

The effects of various poisons are as follows: Briefly there is a predominance of fatty infiltration, a tendency for zonal change (probably related to varying enzyme susceptibility), and damage in several organs other than the liver. Recently it has been shown that the lesion involves destruction of fatty acids making up the smooth endoplasmic reticulum, by the poison or one of its metabolic products (Hashimoto, Glende and Recknagel, 1968). These fatty acids undergo lipid peroxidation, and it is of interest that various factors and certain drugs affect the severity of this process. I have illustrated this in Fig. 2 where I have tried to distinguish between a direct hepatic poison and an indirect one. Carbon tetrachloride is an example of the latter type, for the toxicity of carbon tetrachloride resides in a metabolic breakdown product and not in the poison itself. The production of the toxin will

Fig. 1.

depend on the action of drug-modifying enzymes which one would expect to be diminished in concentration when there is liver disease or protein deficiency; so under these circumstances carbon tetrachloride might be less toxic than in normals. Certainly this is demonstrable in the protein-deficient rat (McLean and McLean, 1966) which becomes relatively resistant to carbon tetrachloride compared with his healthy control. Of course a direct hepatotoxin, that is one that does not depend on metabolic change for the production of liver damage, would react in the opposite way.

Perhaps too one can explain the increased toxicity that is seen clinically when carbon tetrachloride is taken with alcohol. It has been shown (Lieber and Rubin 1968) that alcohol produces hyperplasia of the smooth endoplastic reticulum (SER) as well as liver cell damage. This could then

Fig. 2.

increase the rate of drug breakdown and the liberation of toxic products. This is an example of the most important and interesting phenomenon of enzyme induction. A wide range of commonly used drugs will perform this function, and such drugs will modify their own metabolic rate and the rate at which other drugs and compounds are metabolized.

The sedative drug phenobarbitone is a powerful enzyme-inducer and it has been used clinically in this light to treat some types of jaundice by increasing the rate of glucuronide formation, and therefore the chemical change of bilirubin to bilirubin glucuronide, thus hastening bilirubin excretion. Actually this is almost certainly a gross over-simplification of what happens, as hepatic bilirubin uptake and the so-called ' early peak ' bilirubin are affected by this drug. At any rate SER hyperplasia and increased ability to form glucuronide can be demonstrated in patients such as one described by Whalton, Krustev, and Billin (1968), a woman with unconjugated hyperbilirubinaemia, in whom barbiturate therapy brought about a dramatic decrease of serum bilirubin levels. The search for drugs which are good enzyme-inducers but which do not have unpleasant or unwanted side effects (such as barbiturates have) will go on, for in this way it may be possible to ' stimulate ' metabolism in patients with liver cell failure, and this might have a valuable role in therapy—an example of flogging a dying horse and yet getting it to work harder. Certainly Thompson and William (1967) have used barbiturates in chronic cholestasis, with benefit as regard reduction of jaundice, though the action of barbiturate here must be a complex one and of course there is much in print about the value of barbiturates in the prevention of jaundice of the newborn (Yaffe, Levy, Matsuzawa and Baliah, 1966; Maurer, Wolfe, Finster, Poppers, Pantuck, Kuntzman and Connery, 1968). Perhaps you might wonder whether caffeine or alcohol compares with phenobarbitone as regards potency of enzyme-induction. Unfortunately these are poor inducers, so that alcoholic intoxication is a rather ineffective way of getting the best out of your hepatic enzyme systems; but then so too is coffee drinking (McLean, 1968).

Of the drugs which initiate a hepatitis-like reaction in the liver there is no doubt that the hydrazine types of mono-amine-oxidase inhibitors and the anaesthetic agent halothane have created most interest. In a way the retrospective survey of the National Halothane Study (Sub-committee on National Halothane Study of Committee on Anaesthesia, 1966) which reported on the hepatic complications resulting from 800,000 anaesthetics, tended to show that hepatic damage was no more common after halothane than after other anaesthetics, but that when acute hepatic necrosis did occur after anaethesia without apparent aggravating cause, halothane was more likely to have been the anaesthetic used. The figures issued by the Fulminant Hepatitis Group on 150 cases from 73 centres (Trey, Lipworth, Chalmers, Davidson, Gottlieb, Popper and Saunders, 1968) have however again highlighted the fact that halothane may be a more important cause of this syndrome than was previously suspected. Twenty four per cent of the cases in this survey gave a history of previous administration of halothane before the acute event, and of these patients 77% had had more than one exposure to the agent. Halothane has been shown to be a personal risk to at least two anaesthetists, for after using the agent in their practice they showed evidence of impaired liver function. Of course it is extremely difficult to show any significant difference in hepatotoxicity among drugs of this type because of the low frequency of susceptible patients. The end result of this lesion is perhaps the thing to remember—and this is the very serious disorder of acute hepatic necrosis, a highly lethal condition. It is uncertain how halothane can produce its damaging effect. Perhaps some microsomes are susceptible to its action, and there is a secondary inhibition of drug-metabolizing enzymes so that other agents whose metabolism is altered can produce hepatic dysfunction and damage.

I should now like to consider the metabolism of drugs by the liver. A large number of metabolic changes occur in the liver including oxidation, dehalogenation, de-esterification, reduction, and conjugation. In liver disease one might expect these processes to be performed less rapidly. Certainly cortisol, which normally is changed by hepatic reductase to tetrahydrocortisol prior to conjugation and excretion, undergoes change more slowly in liver disease. There is a resultant slower turn-over to cortisone (both endogenous and that given as treatment in hepatic disease). This explains the extreme

Fig. 3.

susceptibility of patients with liver disease to the side effects of corticosteroids; and in a similar way slow hepatic metabolism of hormones such as oestrogens may explain some of the cutaneous vascular signs that one sees in such patients, including " liver palms " and spider naevi. One must also remember that protein binding may be affected by liver disease and in certain circumstances this may also modify the effect of drugs.

There is a general impression that drug metabolism is slower in liver disease. Fig. 3, taken from the paper by Levi (1967) reminds one of the factors that have to be considered before such statements are accepted. The mean $T\frac{1}{2}$ for the removal of a drug (butazolidine) is shown in the left hand column. The same group has been subdivided into two groups on the right where you will see that there is a marked difference in the clearance rate depending on whether previous drugs (of any sort) have been taken or not; and when other drugs have been administered the $T\frac{1}{2}$ for butazolidine is significantly shorter. This effect is also seen in liver disease, and again we are dealing with drug induction of enzymes. Perhaps this effect is less in liver disease, certainly one might expect less ability of the drug metabolizing systems to proliferate when there is deficient protein supply; but this

Fig. 4.

aspect of drug metabolism needs close examination. I would hasten to point out that drug enzyme-induction is not always beneficial, for it is the basis of phenomena like drug resistance and of course is responsible for the precipitation of acute porphyria when increased ALA-synthetase results from phenobarbitone or alcohol administration.

I should like to turn next to the third aspect of drug actions in liver disease and this concerns the extreme susceptibility of the patient with liver disease to the side effects of drugs. The important complication which they produce is hepatic encephalopathy, and the drugs which are most prone to do this are the diuretics and the sedative drugs. It is not surprising that strong sedative drugs such as morphine or pethidine should have an increased sedative effect in patients with liver disease, though it is still not known whether this effect is due to increased brain susceptibility, or slower metabolism, or both. Using the EEG it is possible to monitor the effects of small and clinically harmless doses of various drugs to see if they have a potentially dangerous effect. One uses the slowing of the EEG to monitor this effect, and it is perfectly possible to make the EEG very sensitive and to quantitate the changes that occur, providing that one has an electronic wave-form analyser which allows one to calculate changes in the mean EEG frequency. Spontaneous changes are normally very small so that a drug-induced shift is identifiable.

Fig. 4 shows the effect of a mild tranquilizer (chlorpromazine) on the EEG mean dominant frequency (MDF) for patients with and without liver disease. There is a highly significant difference in the degree of slowing seen, the liver patients being more susceptible to the chlorpromazine which was given in a dose of 1 mg/Kg i.e. well within the therapeutic dose-range. Though there is a difference in response, the statistical analysis is not satisfactory because of the extreme variability of response to the drug, one or two patients showing a very marked degree of slowing. Analysis of the data of the liver disease group shows that there is a relationship between the degree of the shift of the EEG and a previous history of clinical hepatic encephalopathy, and also the EEG of the patient prior to the drug being given. When we correlate the degree of change with these two factors we find that of six patients who developed a large EEG change as a result of drug therapy, five had had previous episodes of clinical encephalopathy and all had a relatively normal EEG, i.e. the peak activity of the pre-shunt record was eight or more cycles per second in each case. In contradistinction to this, the patients with a slower basal EEG (whether they had had previous encephalopathy or not) showed a smaller EEG change.

Of course it is not unusual for the EEG to be slower than normal in hepatic encephalopathy, though the type of abnormality is nonspecific and is shared by a number of metabolic encephalopathies. With the onset of chronic hepatic encephalopathy and resultant organic damage the EEG becomes slower than normal but also less liable to change with the administration of drugs. Providing this is appreciated, EEG changes of this type are a valuable way of monitoring the effect of drugs.

Though the brain in liver disease may be susceptible to the action of drugs it is perfectly possible for damage to other organs to play a part in explaining other abnormal drug effects. Malabsorption may affect drug absorption and therefore drug action; renal disease may result in drug retention, and at least one renal abnormality which is not infrequently found in cirrhosis (Shear, Bonokowsky, and Gabuzda, 1969), namely renal tubular acidosis of the acquired type may, by increasing renal ammonia production, facilitate the development of hyperammonaemia and encephalopathy, or at least partially explain the fact that certain diuretics will produce a similar effect. Similar abnormalities of function in other organs may explain other abnormalities of drug action.

I have suggested to you that we know next to nothing about drug activity in normal subjects and its genetic variations. We know even less, if that is possible, about drug metabolism in the face of dysfunction in the liver or indeed in any other organ.

### References

Hashimoto, S., Glende, E. A. and Recknagel, R. O. (1968) New Eng. J. Med. *279*, 1082.

McLean, A. E. M. and McLean, E. K. (1966) Biochem. J. *100*, 564.

Lieber, C. S. and Rubin, E. (1968) Amer. J. Med. *44*, 200.

Whalton, M. J., Krustev, L. P. and Billing, B. H. (1968) Amer. J. Med. *45*, 160.

Thompson, R. P. H. and Williams, R. (1967) Lancet *2*, 646.

Yaffe, S. J., Levy, F., Matsuzawa, T. and Baliah (1966) New Eng. J. Med. *275*, 1461.

Maurer, H. M., Wolfe, J. A., Finster, M., Poppers, P. J., Pantuck, E., Knutzman, R. and Connery, A. H. (1968) Lancet *2*, 122.

McLean, A. E. M. (1968) Lancet *2*, 1035.

Subcommittee on National Halothane Study of Committee on Anaesthesia (1966) J.A.M.A. *197*, 775.

Trey, C., Lipworth, L., Chalmers, T. C., Davidson, C. S., Gottlieb, L. S., Popper, H. and Saunders, S. J. (1968) New Eng. J. Med. *279*, 798.

Levi, A. J. (1967) In The Liver Vol. 19 of the Colston Papers. Ed. A. E. Read, p. 165 Butterworths London.

Shear, L., Bonkowsky, H. L. and Gabuzda, G. J. (1969) New Eng. J. Med. *280*, 1.

## Figures and Tables

**Fig. 1. f1:**
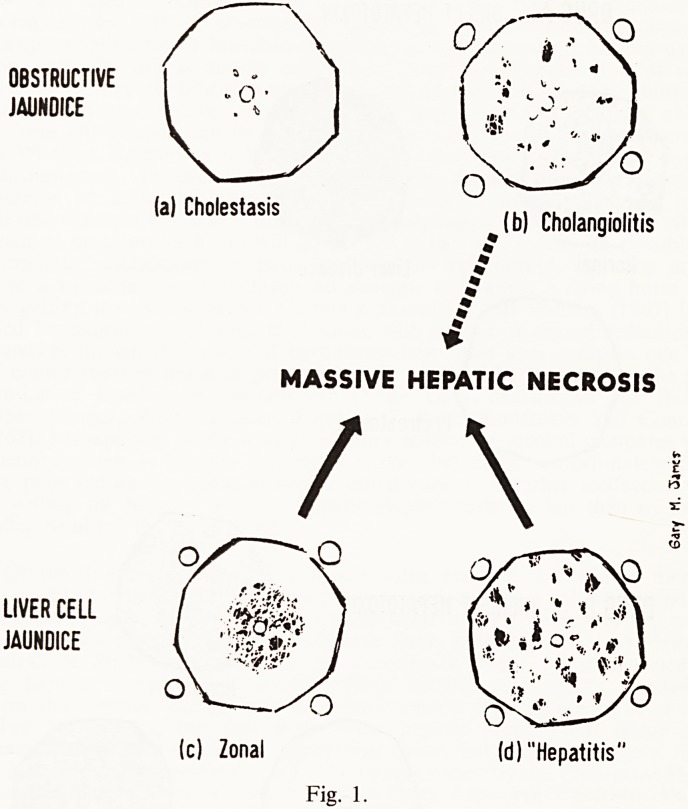


**Fig. 2. f2:**
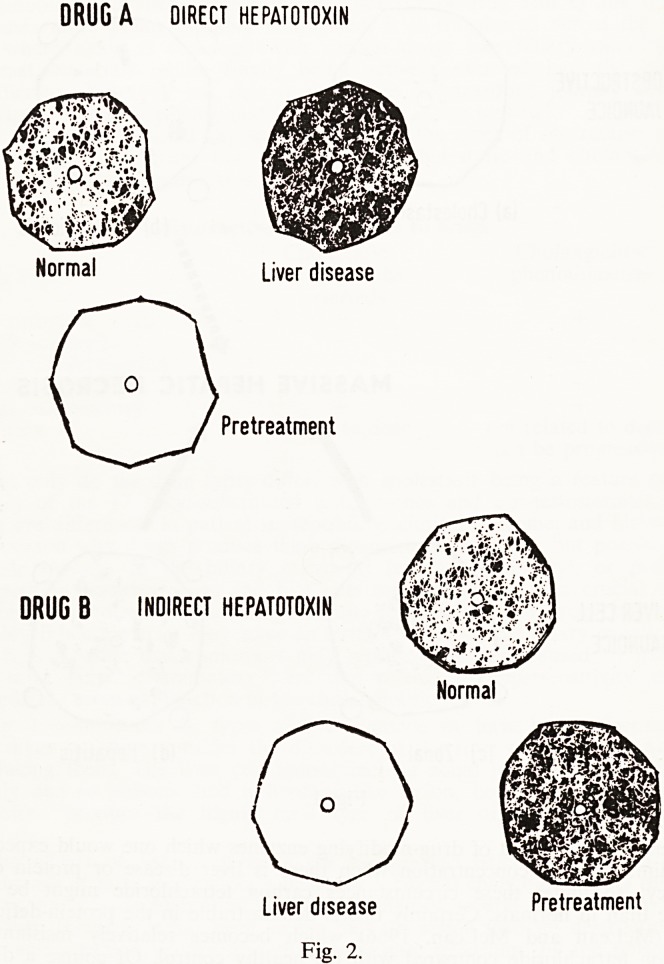


**Fig. 3. f3:**
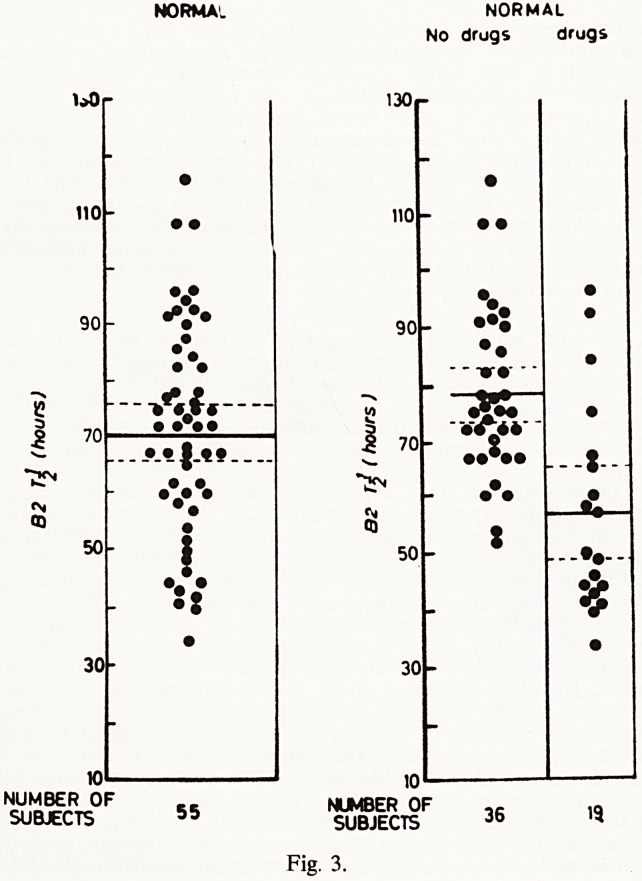


**Fig. 4. f4:**